# Speckle-Based Maximum Density Theory for Micro- and Nanoparticle Characterization via Dynamic Light Scattering

**DOI:** 10.1021/acsomega.5c07734

**Published:** 2025-10-20

**Authors:** Tony M. Silva, Roberta C. A. Oliveira, Ammis S. Álvarez, Wictor C. Magno, Renato Barbosa-Silva, Anderson L. R. Barbosa, José Ferraz

**Affiliations:** † Departamento de Física, 67744 Universidade Federal Rural de Pernambuco, Recife 52171-900, Pernambuco, Brasil; ‡ Escola Politécnica de Pernambuco, 452912 Universidade de Pernambuco, Recife 50720-001, Pernambuco, Brasil

## Abstract

Dynamic light scattering (DLS) is a widely used technique for characterizing suspended micro- and nanoparticles by analyzing their Brownian motion. Here, we introduce Maximum Density Speckle Scattering (MDSS), an alternative approach based on the analysis of dynamic speckle intensity time series. By applying maximum density theory—originally developed for the statistical analysis of nuclear reactions and later adapted for the study of conductance fluctuations—we analyze a time series associated with speckle patterns in DLS experiments to determine the average diameter of silica particles. From the density of maxima on the time series, it is possible to obtain the correlation time and calculate the particles’ diameter. A key advantage of this method is its robustness against optical complexities, as it circumvents the need for explicit optical parameters required in conventional DLS. Using three distinct particle sizes, we demonstrate the method’s efficiency and robustness in the results, even when varying the sample’s concentration, showcasing its potential as a promising alternative for micro- and nanoscale material characterization. The achieved experimental accuracy rivals conventional techniques, demonstrating the potential of MDSS for measuring micro- and nanoparticle dimensions.

## Graphical abstract

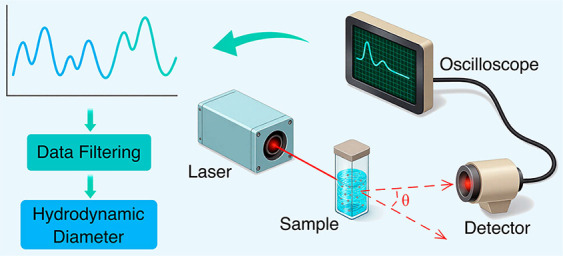

## Introduction

Nanotechnology is a field of science that has been widely explored in recent decades, leading to great interest in the industrial and academic sectors due to its vast application potential.^1^ The possibility of manipulating materials at the nanometric scale has driven significant advances in several areas, such as health, natural sciences, and engineering. In medicine, for example, nanoparticles are used in the diagnosis and treatment of diseases, being applied in imaging exams, such as magnetic resonance imaging and tomography, and in controlled drug release strategies, allowing for increased efficacy and reduced side effects of treatments.^2,3^ In the industrial sector, these structures contribute to the improvement of catalysts,^4^ coatings,^5^ and electronic devices.^6^ Furthermore, its applications cover areas such as the environment,^7^ energy,^8^ and agriculture,^9^ consolidating their position as areas of research in nanotechnology.

The accurate characterization of the morphological properties of micro- and nanoparticles is essential due to the aforementioned applications. Different techniques are applied to estimate distinct parameters, such as size, shape, surface charge, and chemical composition. The choice of the appropriate technique depends on the specific properties of the nanoparticle under study, the desired information, and the limitations of each method. In both academic and industrial research, light scattering techniques are widely applied for characterization of the average diameters of micro- and nanoparticles. Among the most widely used techniques, dynamic light scattering (DLS) stands out.^10^ This technique analyzes the temporal variation in the intensity of scattered light caused by the Brownian motion of particles by measuring the intensity at a fixed angle. The analysis of the temporal dependence of intensity fluctuations, by means of a correlation function, provides the diffusion coefficients of suspended particles. Thus, the average radius or diameter of these particles can be calculated using the Stokes–Einstein equation, which plays a central role in the characterization of particles via DLS.

The diffusion coefficient measures the speed at which suspended particles move in a fluid and can be related to the decay rate or the correlation time of fluctuations in the intensity of scattered light.^11^ In chaotic systems, such as suspended particles, the correlation time becomes an essential parameter as it allows quantification of the degree of disorder in the system. When the movement of particles in a system is unpredictable, the correlation time tends to be shorter, indicating a rapid loss of temporal correlation. In contrast, in systems where particles move in a more orderly and predictable manner, the correlation time is longer, reflecting a longer-lasting correlation over time.^12^

For particles undergoing Brownian motion, the correlation time also varies according to the average diameter of the particles: larger particles lead to longer correlation times, while smaller particles exhibit shorter correlation times.^13^ This results from the speed of movement of the particles in the fluid, where smaller particles tend to move faster and exhibit more disordered behavior compared to larger particles, as they are more affected by the impacts of the fluid molecules. In addition, factors such as the degree of viscosity and the temperature of the fluid can also affect the speed of movement of the particles.^14^ When the correlation function is normalized, that is, with an initial value equal to one, the correlation time can be obtained by measuring the width of the autocorrelation curve at half height.

In a study that analyzes chaotic systems in the context of biodiversity, addressing the chaotic temporal evolution of species over generations,^15^ the authors propose a method that relates the average species population density of maxima to the correlation time. This approach was initially developed for the statistical analysis of nuclear reactions^16^ and, later, expanded to the study of conductance fluctuations.^17–21^ In these studies, the authors demonstrate that from the characteristic correlation function of the system it is possible to define specific relations for the correlation time as a function only of the average density of maxima present in the chaotic time series. This characteristic gives the method flexibility that makes it applicable to a variety of chaotic phenomena in dynamic systems. However, it has not yet been applied in DLS experiments, for which there is significant interest in accurately measuring the correlation time and decay rate of the system.

Accurately estimating the mean diameter of micro- and nanoparticles is crucial for determining their nonlinear optical coefficients, a key parameter in identifying promising materials for all-optical devices.^22^ While electron microscopy techniques—such as transmission (TEM) and scanning electron microscopy (SEM)—provide high-resolution imaging, their high cost and operational complexity limit their accessibility.^23^ In contrast, light scattering techniques provide a cost-effective and simpler to implement alternative for particle characterization. Developing reliable, low-cost methods to estimate the particle diameter is therefore essential to accelerate the characterization process and facilitate broader applications in materials science and photonic device engineering.

This work presents a proof of principle for the maximum density speckle scattering (MDSS), an application of the density of maxima technique in the context of a DLS experiment,^24^ thus offering a new approach for the characterization of micro- and nanoparticles. MDSS is based on a statistical method—previously applied to chaotic systems in nuclear physics and biodiversity—to DLS, enabling robust particle sizing through the analysis of dynamic speckle intensity time series. Three silica nanoparticle samples of distinct sizes were employed as scattering centers in a DLS experiment. To benchmark our proposed characterization method, we compared our results with initial analyses using established techniques, conventional DLS and TEM, both recognized for reliable nanoparticle sizing.^25,26^ While the first two samples were characterized, the third sample’s dimensions were approximated from synthesis stoichiometry.^27^ Our results achieve precision comparable to conventional techniques, highlighting MDSS as a viable tool for micro- and nanoparticle dimensional analysis.

## Theory

### DLS Technique

When a particle is exposed to a light source, such as a laser, it can interact with the light beam in several ways, including absorption, reflection, refraction, or a combination of these three phenomena simultaneously.^28^ These interactions can occur at different levels of intensity depending on the optical properties of the particle and the characteristics of the incident beam. In the DLS technique, which is also known as photon correlation spectroscopy (PCS), we have the presence of a photodetector responsible for capturing the light that is scattered in its direction. The variation in the light intensity is recorded as a function of time at sufficiently short intervals. The analysis of the fluctuations in the signal intensity allows us to autocorrelate it in time. From the temporal autocorrelation function, it becomes possible to calculate the decay rate Γ­(s^–1^), which is correlated to the diffusion coefficient *D* of the particles and the correlation time τ_c_(s).^11^ In DLS, the mean light intensity can be taken as a temporal average, given by

$$\langle I\rangle =\lim_{T\to \infty }\;\frac{1}{T}\int_{0}^{T}I(t)dt$$ 1

where the normalized autocorrelation function is defined as

$$g^{\left(2\right)}\left(\tau \right)=\frac{\left\langle I\left(t\right)I\left(t+\tau \right)\right\rangle }{{\left\langle I\left(t\right)\right\rangle }^{2}}$$ 2

with *T* representing the total duration of the observation, *I*(*t*) and *I*(*t* + τ) correspond to the intensities at instants *t* and *t* + τ, respectively.^10,11^

Considering that the signal collected by the photodetector can be converted into electrical pulses, eq 2 is related to an electric field correlation function *g* ^(1)^(τ) through the Siegert relation:^29^

$$g^{\left(2\right)}\left(\tau \right)=1+|g^{\left(1\right)}\left(\tau \right){|}^{2}$$ 3

In some cases, a factor β ≤ 1 is added to account for contrast reduction as the field or intensity is averaged over speckles or uncorrelated modes:^30^

$$g^{\left(2\right)}\left(\tau \right)=1+\beta |g^{\left(1\right)}\left(\tau \right){|}^{2}$$ 4

Experimentally, β = 1 means that the system is configured in such a way that all captured fluctuations are coherent with no loss of contrast in the correlation function. A laser with high temporal and spatial coherence provides a high contrast, which allows us to assume β = 1.

When the system is monodisperse, the function *g* ^(1)^(τ) is described by a simple exponential decay, given by

$$g^{\left(1\right)}\left(\tau \right)=\exp\left(-\Gamma \tau \right)$$ 5

where τ represents the time interval in which the correlations are established, and the parameter Γ denotes the previously mentioned decay rate.^31^ However, in polydisperse systems, the autocorrelation function reflects a mixture of several decays, with each one corresponding to different particle sizes. In this context, different methods suggest that adjustments to the correlation function *g* ^(1)^(τ), allowing the separation of the different decays. One of the best known is the cumulant method, which suggests the following expression for the electric field correlation function:

$$g^{\left(1\right)}\left(\tau \right)=\exp\left(-\left\langle \Gamma \right\rangle \tau \right)\left(1+\frac{\mu_{2}}{2!}\tau^{2}+\frac{\mu_{3}}{3!}\tau^{3}+\cdot \cdot \cdot \right)$$ 6

where μ_*n*_ represents the moments or cumulants of the expansion, and ⟨Γ⟩ is the average decay rate of the system.^11,31^ Regardless of the type of system, whether monodisperse or polydisperse, the relationship between the decay rate and diffusion coefficient *D* is given by the following expression:

$$\Gamma =Dq^{2}=\tau_{c}^{-1}$$ 7

In this expression, *q* is the magnitude of the scattering vector,^10^ which is defined as the difference between the wave vector of the incident beam and that of the scattered beam, and its magnitude is given by

$$q=\frac{4\pi n}{\lambda }\sin\left(\frac{\theta }{2}\right)$$ 8

where *n* is the refractive index of the solvent, λ is the wavelength of the incident beam, and θ is the scattering angle. Furthermore, given that the correlation time τ_c_ is inversely proportional to the decay rate,^11^ it is also related to the diffusion coefficient as shown in eq 7.

Once the decay rate of the system and the magnitude of the scattering vector are known, the diffusion coefficient is determined as shown by eq 7. As a result, it becomes possible to estimate the radius or average diameter of suspended particles, assuming that they are spherical, identical, and noninteracting. This estimate is obtained by applying the Stokes–Einstein equation:^32^

$$D=\frac{k_{B}T}{6\pi \eta r}$$ 9

where *k*_B_ is the Boltzmann constant, *T* represents the absolute temperature of the medium, η corresponds to the dynamic viscosity of the fluid where the particles are suspended, and *r* denotes the radius of the suspended particles.

### Density of Maxima

In the studies developed by Bazeia et al.^15^ and Medeiros et al.,^12^ the authors present a technique to determine the correlation time from the analysis of the average density of maxima in systems that exhibit chaotic behavior. This approach was originally developed for the statistical analysis of nuclear reactions,^16^ and later extended by Ramos et al.^17^ to analyze conductance fluctuations in chaotic quantum dots. More recently, it was successfully applied to the analysis of experimental data on universal conductance fluctuations in quasi-one-dimensional nanowires.^20,21^ Here, we establish a relationship between the correlation time and the density of maxima observed in the intensity time series of a dynamic speckle scattering experiment, which is the theoretical aspect of the MDSS method.

To introduce this subject, let us consider the existence of a variable *I*, whose temporal evolution is subject to a fluctuation time to produce a local maximum in the interval [*t*, *t* + τ], for a sufficiently small τ, that is, τ → 0. Approaching a maximum point from the left side, the derivative of *I* is expected to be positive, *I′*(*t*) > 0, and from the right side negative, *I′*(*t* + τ) < 0. Furthermore, in order to have a downward concavity, the second derivative is expected to be negative, *I*″(*t*) < 0, which leads us to −*I*″(*t*) τ > *I′*(*t*) > 0.^15^

To calculate the average density of maxima ⟨ρ⟩, we can use the joint probability *P*(*I′*, *I*″). Considering that the probability of finding a maximum in the interval [*t*, *t* + τ] is proportional to the integral that covers the entire defined region, we will have that

$$\left\langle \rho \rangle ≡\frac{1}{\tau }\int_{-\infty }^{0}dI^{\prime \prime }\int_{0}^{-I\prime \prime \tau }dI^{\prime }P(I^{\prime },\;I^{\prime \prime })=-\int_{-\infty }^{0}dI^{\prime \prime }I^{\prime \prime }P(0,\;I^{\prime \prime }\right)$$ 10

Considering the fact that the statistical properties of the mean number of peaks are time-invariant, both *I′* and *I*″ will have zero mean values.^12^ Furthermore, the properties of *P*(*I′*, *I*″) can be obtained from the smallest moments of *I′* and *I*″, while the variances of *P*(*I′*, *I*″) are directly related to the correlation function:^15^

C(τ)=⟨I(t)I(t+τ)⟩ 11

The moments of *I′* and *I*″ can be obtained from their derivatives, which are

$$\langle I^{\prime 2}\rangle =-\frac{d^{2}C(\tau )}{d\tau^{2}}{|}_{\tau =0}\;\mathrm{and}\;\langle I^{\prime \prime 2}\rangle =\frac{d^{4}C(\tau )}{d\tau^{4}}{|}_{\tau =0}$$ 12

To construct the joint probability distribution for *I*(*t*) and its derivatives, the principle of maximum entropy can be applied. After performing the necessary algebraic calculations, integration with respect to *I*(*t*) yields *P*(*I′*, *I*″), which gives us:^15^

$$P(0,\;I^{\prime \prime })=\frac{1}{2\pi }\frac{1}{\sqrt{\left\langle I^{\prime 2}\rangle \langle I^{\prime \prime 2}\right\rangle }}\exp\left(-\frac{1}{2}\frac{I^{\prime \prime 2}}{\left\langle I^{\prime \prime 2}\right\rangle }\right)$$ 13

By substituting this expression into eq 10 and solving the integral in terms of *I*″, we will obtain

$$\langle \rho \rangle =\frac{1}{2\pi }\sqrt{\frac{\left\langle I^{\prime \prime 2}\right\rangle }{\left\langle I^{\prime 2}\right\rangle }}$$ 14

This is an extremely important result, allowing us to determine different relations to obtain the correlation time, τ_c_, as a function of the average density of maxima, ⟨ρ⟩. These relations will arise according to the application context and its characteristic correlation function. For example, in the study presented in ref 17, the authors determined two distinct expressions for the correlation time, $\tau_{c}=\sqrt{3}/(\pi \langle \rho \rangle )$ and $\tau_{c}=3/(\pi \sqrt{2}\langle \rho \rangle )$ , obtained from different correlation functions: a simple Lorentzian and a square Lorentzian. In ref 15, another relation is presented, τ_c_ = 1/(6⟨ρ⟩), derived from another correlation function that has an oscillatory behavior.

Since the correlation time, τ_c_, is related to the diffusion coefficient, *D*, by means of eq 7, the density of maxima technique can be applied together with the Stokes–Einstein equation to determine the mean diameter of suspended particles. For this purpose, a new relation for τ_c_ as a function of ⟨ρ⟩, specifically aimed at applications in DLS experiments, is presented. This relation was derived from the correlation function described by eq 3; however, when using the value of *g* ^(1)^(τ) given by eq 5, we found the presence of an imaginary factor in the final expression of the average maximum density obtained from eq 14, which makes the use of the function *g* ^(1)^ = exp­(−Γτ) unfeasible.

To address this limitation, we considered alternative methods capable of providing a suitable first-order correlation function, *g* ^(1)^(τ), particularly those derived from experimental configurations analogous to DLS. A relevant theoretical-experimental study on dynamic speckle formation^24,33^ offers a statistical framework for this phenomenon, including a derived expression for *g* ^(1)^(τ) expressed exclusively in terms of spatial and temporal parameters:

$$|g^{\left(1\right)}(\tau ){|}^{2}=B\exp\left(-\frac{\tau^{2}}{\tau_{c}^{2}}\right)$$ 15

where *B* = exp­(−*l* ^2^/*l*_0_^2^), and the terms *l* and *l*_0_ correspond to the spatial detection range and the mean size of the speckles projected onto the observation plane, respectively. In our application case, these terms will be interpreted as constants since the values of ⟨*I′* ^2^⟩ and ⟨*I*″^2^⟩ will be obtained from the time derivatives of *g* ^(2)^(τ). Therefore, by substituting eq 15 into eq 3, we will obtain

$$g^{\left(2\right)}\left(\tau \right)=1+B\exp\left(-\frac{\tau^{2}}{\tau_{c}^{2}}\right)$$ 16

and consequently, by substituting eq 16 into eq 14, we will have

$$\tau_{c}=\frac{\sqrt{6}}{2\pi \langle \rho \rangle }$$ 17

The application of eq 15, deduced from the formation of dynamic speckles, can be justified in DLS experiments by taking into account the similar nature of the light scattering phenomena present in both cases. As discussed previously, in DLS, a beam of light is scattered by suspended particles, creating an interference pattern that results in temporal fluctuations in the intensity of the detected signal. These fluctuations are analogous to the variations observed in the formation of dynamic speckles, where light is scattered by an irregular surface or by moving particles.^24,33,34^ Eq 17 is the expression that characterizes, from the theoretical point of view, the MDSS method. Although we have deduced a new relation for the MDSS case, tests were performed specifically with the relations $\tau_{c}=\sqrt{3}/(\pi \langle \rho \rangle )$ and $\tau_{c}=3/\left(\pi \sqrt{2}\left\langle \rho \right\rangle \right)$ since the correlation curve obtained experimentally in these studies resembles the Lorentzian form, suggesting a possible application in DLS experiments.

However, the results obtained from these expressions deviated significantly from expected values. In some cases, the estimated hydrodynamic diameters were nearly double those measured by conventional DLS and TEM techniques. Comparing the relationships $\tau_{c}=\sqrt{3}/(\pi \langle \rho \rangle )$ and $\tau_{c}=3/(\pi \sqrt{2}\langle \rho \rangle )$ , with the expression proposed for the MDSS, $\tau_{c}=\sqrt{6}/(2\pi \langle \rho \rangle )$ , we observe that the two former equations yield slightly longer correlation times, explaining the overestimated values. In this way, the expression developed in this work proved to be more suitable for DLS applications, providing estimates closer to the results obtained by the established methods. This performance was expected, as the equation was specifically derived for this purpose, accounting for the particularities and characteristics of the physical system involved in the present investigation.

It is of fundamental importance to note that the MDSS technique circumvents the necessity for explicit optical parameters such as refractive index, wavelength, and detector’s angle, a requisite that is a limitation of conventional DLS methods that rely on the absolute determination of the scattering vector *q*. By analyzing the temporal density of maxima, which is directly proportional to the decay rate Γ, or inversely proportional to the coherence time τ_c_, MDSS inherently absorbs the collective optical parameters into a fixed proportionality constant for a given instrument configuration. Consequently, particle size is determined from the fluctuation’s “clock speed”—the characteristic rate or frequency at which the scattered light intensity signal fluctuates due to Brownian motion—rather than an isolated *q*_2_ term, rendering the absolute value of *q* irrelevant and establishing a robust advantage for measurements within complex optical environments.

## Materials and Methods

### Samples and Preliminary Characterization

For this study, we employed three samples of silica nanoparticles with different average diameters, synthesized according to the procedure described in ref 27. After the synthesis, these samples were diluted in ethanol. The dimensions of the first two samples were previously determined through DLS and TEM techniques, using an NPA 152-32A Zetatrac (Microtrac) and an FEI Tecnai20 200 kV electron microscope, respectively. The third sample had unknown dimensions, but they could be estimated based on the volume of reagents used in its synthesis, such as ammonium hydroxide (NH_4_OH), whose influence on the final particle size was clearly demonstrated in ref 27. Thus, in addition to TEM, it will also be possible to compare the approach presented in this study to the approach commonly used by commercially available DLS equipment.

Table 1 presents the particle concentration per volume of ethanol (parts/mL), the average diameter of each sample, and the amount of NH_4_OH used in the synthesis process. As can be observed, only the first sample had its diameter estimated by both techniques, TEM and conventional DLS. When it comes to DLS, testing different dilutions during the analysis process is extremely common and recommended to obtain reliable results. For the analysis of the first and second samples, S1 and S2, volumes of 200 and 100 μL, respectively, were taken. Both samples were initially diluted in 2 mL of ethanol, and gradually, measurements were taken with slightly higher dilutions until the same average diameter corresponding to each sample was obtained, regardless of the dilution used. For TEM analysis, a volume of 100 μL of S1 was diluted in an additional 8 mL of ethanol, in which approximately 1000 particles were evaluated to ensure a representative estimate of the average particle diameter of S1. Figure 1a shows an image of the silica nanoparticles, and Figure 1b presents a histogram for the diameter of sample S1, obtained during the TEM characterization process.

**Table 1:** Previously Estimated Diameters for Each of the Silica Samples^a^

sample	NH_4_OH [mL]	diameter NH_4_OH [nm]	diameter DLS [nm]	diameter TEM [nm]	concentration [part./mL]
S1	4.00		146 ± 28	122 ± 50	6.80 × 10^12^
S2	4.75		226 ± 50		1.20 × 10^12^
S3	5.75	≈300			

a The second column shows the volume of NH_4_OH used in the synthesis of each sample, while the third, fourth, and fifth columns indicate, respectively, the estimated diameter based on this volume and the diameters obtained by conventional DLS and TEM techniques. Only sample S1 had its diameter determined by both techniques, whereas for sample S3, the value was estimated solely based on the amount of NH_4_OH used in its synthesis. The last column provides an approximate estimate of the number of particles per volume of ethanol in each sample.

**Figure 1:**
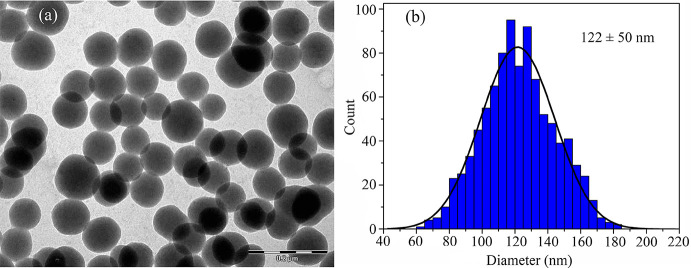
(a) Image of the nanoparticles from sample S1 captured during the TEM characterization process. (b) Histogram of the diameters of the analyzed particles. Approximately 1000 particles were evaluated to ensure a representative estimate of the average particle diameter, resulting in a diameter of 122 ± 50 nm.

### Experimental Setup

The experimental setup consisted of a Melles Griot helium–neon (He–Ne) laser with a central wavelength of 632 nm and a power of 1.6 mW, a Thorlabs photodetector (model APD110A), and a Tektronix MSO2024B oscilloscope with a resolution of 200 MHz and a sampling rate of 1 GSa/s. It is important to note that, although a single-point photodetector is used without spatial resolution, the measured intensity fluctuations are a direct result of the temporal evolution of the dynamic speckle pattern formed by the scattered light.

Additionally, a Branson ultrasonic bath device (model 200) was used to avoid the presence of silica nanoparticle aggregates during data acquisition. Considering that the samples had already been diluted in ethanol, we also opted to use the same type of material to prepare new dilutions for the subsequent analysis process. The ethanol used had a purity of 96%, and a viscosity coefficient of 1.2 mPa ·s was assumed for a temperature of approximately 20 °C.^35,36^

The typical experimental setup for a DLS experiment has a relatively simple configuration. The angle at which the photodetector is positioned relative to the incident light beam is pivotal for proper detection of the light scattered by the silica nanoparticles. Based on previous studies,^37,38^ an initial configuration was adopted where the photodetector was positioned at 90° relative to the incident beam. However, due to the low intensity of the scattered signal detected, this configuration was discarded. In order to improve signal detection, angles between 20 and 40° were tested, varying in intervals of 5°. During the tests, an angle of 20° proved to be the most adequate, allowing the capture of a more intense signal. Figure 2 illustrates the final experimental setup used in this study. It is worth noting that, to prevent external interference, such as ambient light, a black box was built to enclose the sample and the detector. Although the laboratory lights remained off during data acquisition, the detector’s extreme sensitivity still allowed it to capture nonvisible photons, making its isolation and the rigorous minimization of any external sources of interference essential.

**Figure 2:**
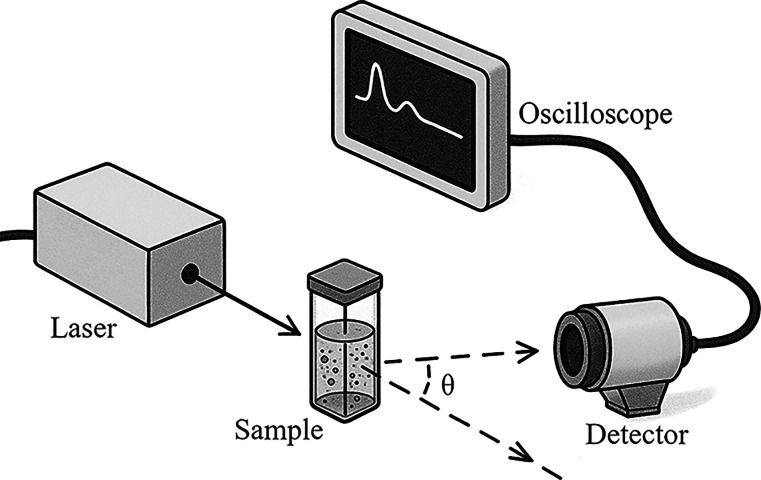
Illustration of the experimental setup used, consisting of a Melles Griot He–Ne laser with a wavelength of 632 nm, a Thorlabs APD110A photodetector positioned at an angle of θ = 20° relative to the incident beam, and a Tektronix MSO2024B oscilloscope with a resolution of 200 MHz and a sampling rate of 1 GSa/s for data reading and storage.

It is important to note that although a single-point photodetector is used without spatial resolution, the measured intensity fluctuations are a direct result of the temporal evolution of the dynamic speckle pattern formed by the scattered light.

### Sample Preparation

Considering that both the diameters and the number of particles varied between the samples, tests with different volumes of ethanol were conducted to estimate the most suitable concentrations for each sample. These tests allowed the identification of concentrations that produced the maximum scattering possible without compromising the results since a highly concentrated sample could lead to the rapid formation of agglomerates, resulting in scattering and hindered diffusion, leading to inaccurate data analysis. Similarly, an insufficient number of particles per volume could hinder the correlation of the scattered light signal with the average particle diameter.

Another relevant aspect to consider is the significant presence of noise in the data. When analyzing the autocorrelation graph of the signal, an abrupt initial decay in the autocorrelation curve was observed, indicating the presence of noise. However, while testing with specimens with a low concentration for sample S1, it was noticed that the amount of noise in the data also decreased as less ethanol was used to prepare the concentrations. This occurred because more particles per volume could interact simultaneously with the beam, which contributes not only to the collection of more data from the scattered light but also to the detection of a more intense signal. However, there is a limit to this effect. As increasingly less diluted concentrations were tested, a gradual increase in noise levels was again observed, suggesting the existence of an optimal threshold to minimize noise through concentration adjustments. The same behavior was observed for the other samples. For sample S2, a larger dilution volume was required to achieve noise levels similar to those of sample S1 since S2’s particles have a larger diameter and can scatter more light. Regarding sample S3, which has the largest diameter among the three, the initial volume of 100 mL of ethanol for 1 mL of sample proved to be adequate, resulting in the lowest noise levels.

Table 2 contains all the concentrations tested for each sample, highlighting (asterisks) those that proved to be most suitable during the tests. These concentrations allowed the acquisition of data with the highest intensity levels and the lowest possible noise, considering the experimental conditions and the available equipment. In the concentration column, the values on the left correspond to the volume of silica, while the values on the right indicate the volume of ethanol used (silica:ethanol). At this stage, it is crucial to accurately measure the temperature of the concentrations, as this is an essential parameter for subsequent analysis, given that temperature directly influences the behavior of particles in the fluid. For data collection and analysis, all tested concentrations were subject to an ultrasonic bath for at least 10 min, after which only a small volume of the solution was transferred to the cuvette.

**Table 2:** Tested Concentrations for Each Sample, with an Asterisk (*) Highlighting Those That Exhibited the Highest Intensity Levels and the Lowest Noise in the Data^a^

samples	concentrations tested (mL)				
S1	1:100	1:80	1:70	*1:60	1:50
S2	1:100	*1:80	1:70	1:60	1:50
S3	*1:100	1:80	1:70		

a In the concentration column, the values on the left correspond to the volume of silica, while those on the right indicate the volume of ethanol used in the mixture (silica:ethanol).

### Data Collection, Processing, and Analysis

To minimize the electronic noise generated by the equipment itself, the noise filtering function available on the oscilloscope was used. This feature allows the configuration of a cutoff frequency that when properly adjusted, significantly reduces unwanted background noise. Among the available options, the frequency of 1.4 kHz was the most suitable for the scattering profile of the silica samples, providing a significant noise reduction without compromising the integrity of the signal.

For data processing and analysis, Wolfram Mathematica software was used, where a script was developed to apply a band-pass filter, complementing the filtering performed by the oscilloscope and eliminating residual noise still present in the signal. Among the various types of filters available, the band-pass filter was chosen due to its operational characteristics. In the context of scattering signal analysis, applying this filter is essential to isolate the frequency range of interest, minimizing unwanted noise that could compromise data analysis.

The analysis of the autocorrelation function of the scattering signal was essential to determining the appropriate adjustment of the band-pass filter. The filtering range was iteratively adjusted until the autocorrelation curve exhibited a decay approaching zero. Before filtering, the curve showed a more prolonged decay and, in some cases, did not reach zero due to the still significant presence of noise. With proper adjustment, these interferences were minimized, ensuring a more accurate and representative autocorrelation curve of the actual signal behavior. In Figure 3a, the signals before and after filtering are presented, where a reduction in signal intensity can be observed due to the effect of the band-pass filter. This effect results from the filter’s capacity not only to attenuate electronic noise but also to reduce background noise, leading to a decrease in signal intensity levels. Figure 3b illustrates the filter’s action throughout the data set, where small noise-induced peaks are eliminated. Finally, Figure 3c displays the autocorrelation curves of both signals, highlighting the more prolonged decay in the detected signal’s curve compared to the filtered signal. This difference arises from the significant presence of noise in the raw data, which distorts the signal behavior and artificially extends the decay of the autocorrelation curve. The small oscillations present on the autocorrelation curves of filtered and unfiltered signals are likely to represent artifacts introduced by the finite impulse response (FIR) of the band-pass filter. Such oscillatory behavior typically arises at frequencies near the filter cutoffs, particularly in band-pass filters where the high-pass and low-pass cutoffs interact.^39^ However, for DLS applications and for the MDSS technique, the analysis is restricted to the short-lag region for determining the correlation time; thus, oscillations at extended lags can be disregarded.

**Figure 3:**
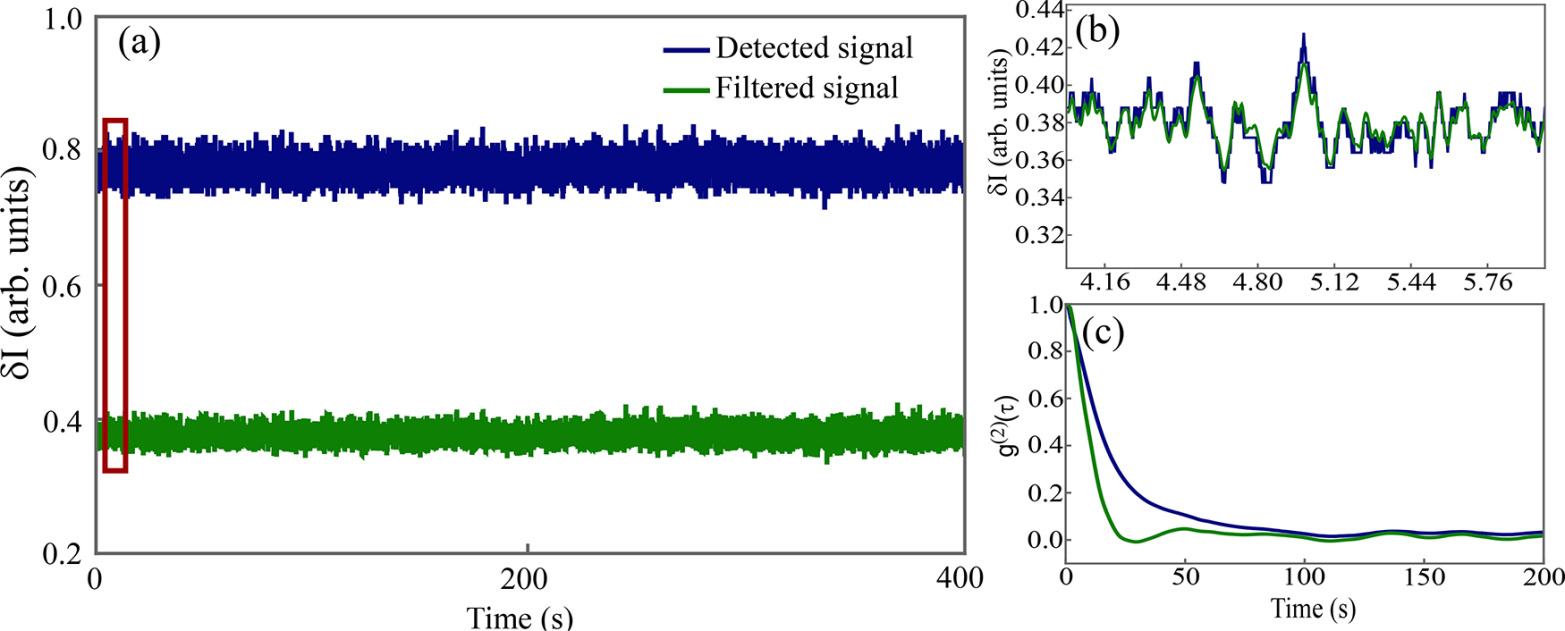
Scattering signal before (blue curves) and after (green curves) the application of the band-pass filter. (a) Comparison between the signals before and after filtering. (b) Filter action along the signal. (c) Autocorrelation curves of both signals.

## Results and Discussion

Using the concentrations highlighted in Table 2, ten consecutive measurements were performed for each sample, with each measurement lasting approximately 6 min, totaling about 1 h for the complete data acquisition. The calculation of the average sample diameter was carried out using eqs 7, 9, and 17, which together allowed us to express the Stokes–Einstein equation in terms of the average density of maxima:

$$d_{h}=\frac{k_{B}Tq^{2}}{\sqrt{6}\pi^{2}\eta \langle \rho \rangle }$$ 18

The term *d*_h_ refers to the hydrodynamic diameter of suspended particles, which accounts not only for the effective particle diameter but also for the additional layer of ions formed around them due to interactions with the dispersion medium. In the case of spherical symmetry, the hydrodynamic diameter can be approximated as the effective particle diameter. Eq 18 describes the hydrodynamic diameter of suspended particles in the MDSS method.

In order to identify the minimum time required for a reliable and accurate estimation of the nanoparticle diameter, the average density of maxima was calculated incrementally. Initially, an arbitrary interval of 10 time steps from the data set was considered, with successive additions of 10 time steps per iteration. The density of maxima was derived for each interval, and the associated diameter was subsequently determined. To compare the results obtained before and after applying the band-pass filter, the values calculated from these two cases will be presented.

Tables 3–5 present the MDSS results obtained for each analyzed sample. In all tables, the terms ⟨ρ_o_⟩ and ⟨ρ_f_⟩ represent the average densities of maxima before and after the application of the band-pass filter, respectively. The corresponding correlation times are denoted by τ_c_(⟨ρ_o_⟩) and τ_c_(⟨ρ_f_⟩), while the terms *d*_h_(⟨ρ_o_⟩) and *d*_h_(⟨ρ_f_⟩) refer to the hydrodynamic diameters estimated from these values. Mean values calculated for each of the listed parameters are shown in the final line of each table.

**Table 3:** MDSS Results Obtained for Sample S1, Diluted at a Concentration of 1:60 mL (Silica:Ethanol)^a^

sample S1, concentration 1:60 [mL]						
measures	⟨ρ_o_⟩ [s^–1^]	⟨ρ_f_⟩ [s^–1^]	τ_c_(⟨ρ_o_⟩) [s]	τ_c_(⟨ρ_f_⟩) [s]	*d*_h_(⟨ρ_o_⟩) [nm]	*d*_h_(⟨ρ_f_⟩) [nm]
M01	35.240	24.025	0.0111	0.0162	81.7	119.9
M02	34.045	23.787	0.0115	0.0164	84.6	121.1
M03	33.727	23.715	0.0116	0.0164	85.4	121.5
M04	33.190	23.530	0.0117	0.0166	86.8	122.4
M05	32.952	23.335	0.0118	0.0167	87.4	123.4
M06	32.655	23.312	0.0119	0.0167	88.2	123.5
M07	32.427	22.880	0.0120	0.0170	88.8	125.9
M08	36.627	22.710	0.0106	0.0172	78.6	126.8
M09	38.297	23.070	0.0102	0.0169	75.2	124.8
M10	39.752	23.235	0.0098	0.0168	72.4	124.0
Average	34.891	23.360	0.0112	0.0167	82.9	123.3

a The terms ⟨ρ_o_⟩ and ⟨ρ_f_⟩ correspond to the average densities of maxima before and after the application of the band-pass filter, respectively. The associated correlation times are represented by τ_c_(⟨ρ_o_⟩) and τ_c_(⟨ρ_f_⟩), while the hydrodynamic diameters estimated from these values are denoted by *d*_h_(⟨ρ_o_⟩) and *d*_h_(⟨ρ_f_⟩). Averaged values calculated for each of the listed parameters are presented at the bottom of the table.

**Table 4:** MDSS Results Obtained for Sample S2, Diluted at a Concentration of 1:80 mL (Silica:Ethanol)^a^

sample S2, concentration 1:80 [mL]						
measures	⟨ρ_o_⟩ [s^–1^]	⟨ρ_f_⟩ [s^–1^]	τ_c_(⟨ρ_o_⟩) [s]	τ_c_(⟨ρ_f_⟩) [s]	*d*_h_(⟨ρ_o_⟩) [nm]	*d*_h_(⟨ρ_f_⟩) [nm]
M01	23.125	12.340	0.0169	0.0316	124.6	233.5
M02	20.202	12.647	0.0193	0.0308	142.6	227.8
M03	29.512	12.137	0.0132	0.0321	97.6	237.3
M04	28.765	12.095	0.0136	0.0322	100.1	238.2
M05	28.565	12.107	0.0136	0.0322	100.8	237.9
M06	27.510	12.120	0.0142	0.0322	104.7	237.7
M07	27.105	12.087	0.0144	0.0323	106.3	238.3
M08	26.760	12.025	0.0146	0.0324	107.6	239.6
M09	27.230	12.142	0.0143	0.0321	105.8	237.2
M10	26.837	12.057	0.0145	0.0323	107.3	238.9
Average	26.561	12.176	0.0149	0.0320	109.7	236.6

a The terms ⟨ρ_o_⟩ and ⟨ρ_f_⟩ correspond to the average densities of maxima before and after the application of the band-pass filter, respectively. The associated correlation times are represented by τ_c_(⟨ρ_o_⟩) and τ_c_(⟨ρ_f_⟩), while the hydrodynamic diameters estimated from these values are denoted by *d*_h_(⟨ρ_o_⟩) and *d*_h_(⟨ρ_f_⟩). Averaged values calculated for each of the listed parameters are presented at the bottom of the table.

**Table 5:** MDSS Results Obtained for Sample S3, Diluted at a Concentration of 1:100 mL (Silica:Ethanol)^a^

sample S3, concentration 1:100 [mL]						
measures	⟨ρ_o_⟩ [s^–1^]	⟨ρ_f_⟩ [s^–1^]	τ_c_(⟨ρ_o_⟩) [s]	τ_c_(⟨ρ_f_⟩) [s]	*d*_h_(⟨ρ_o_⟩) [nm]	*d*_h_(⟨ρ_f_⟩) [nm]
M01	22.665	8.697	0.0172	0.0448	127.1	331.2
M02	22.527	8.857	0.0173	0.0440	127.9	325.3
M03	23.047	8.957	0.0169	0.0435	125.0	321.6
M04	22.615	8.937	0.0172	0.0436	127.4	322.3
M05	22.477	8.902	0.0173	0.0438	128.1	323.6
M06	22.055	8.940	0.0177	0.0436	130.6	322.3
M07	21.145	8.942	0.0184	0.0436	136.2	322.2
M08	20.970	8.847	0.0186	0.0441	137.4	325.6
M09	20.865	8.917	0.0187	0.0437	138.0	323.1
M10	20.325	8.840	0.0192	0.0441	141.7	325.9
Average	21.869	8.884	0.0179	0.0439	131.9	324.3

a The terms ⟨ρ_o_⟩ and ⟨ρ_f_⟩ correspond to the average densities of maxima before and after the application of the band-pass filter, respectively. The associated correlation times are represented by τ_c_(⟨ρ_o_⟩) and τ_c_(⟨ρ_f_⟩), while the hydrodynamic diameters estimated from these values are denoted by *d*_h_(⟨ρ_o_⟩) and *d*_h_(⟨ρ_f_⟩). Averaged values calculated for each of the listed parameters are presented at the bottom of the table.

Initial analysis for the MDSS results in Table 3, corresponding to the first sample (S1) diluted at a concentration of 1:60 mL (i.e., 1 mL of the initial silica sample in 60 mL of ethanol), reveals a significant difference between the mean values of the density of maxima before and after application of the band-pass filter. This difference was expected as the filtering process eliminates small peaks that are abundantly generated by noise and significantly influence the final value of the density of maxima. Regarding the correlation times, although the values calculated before and after filtering show no substantial differences at first glance, their impact becomes more evident in the estimated values for the average particle diameter. Prior to filter application, an average diameter of 82.9 nm was estimated, whereas after filtering, a value of 123.3 nm was obtained using the MDSS method—the latter being in close agreement with the TEM-estimated value (see Figure 1).

The same behavior is observed for the second sample (S2), the MDSS results of which are presented in Table 4. In this case, the difference between the estimated values before and after the application of the band-pass filter is even more pronounced. When the average densities of maxima are compared, a discrepancy of approximately 14 s^–1^ is noted, evidencing the strong influence of noise on the unfiltered data. This difference affects the correlation times and, even more significantly, the hydrodynamic diameters: before filtering, the estimated value was 109.7 nm, whereas after filter application, a value of 236.6 nm was obtained. The latter value closely approximates that obtained by the conventional DLS technique, reinforcing the efficacy of the filtering process in removing high-frequency noise capable of distorting the analysis.

This behavioral pattern is also observed in the MDSS results of the third sample (S3), as shown in Table 5. The estimated average densities of maxima before and after filtering exhibit a difference of approximately 13 s^–1^, which again impacts the correlation times and, consequently, the mean particle diameter values. After filter application, a mean diameter of 324.3 nm was obtained—a value that also demonstrates good agreement with the theoretical estimate of approximately 300 nm, calculated based on the volume of NH_4_OH used in the sample synthesis.^27^ These results highlight that the band-pass filter not only corrects noise-induced deviations but also enables precise estimates consistent with the reference experimental parameters.

Additionally, all of the steps of the new technique proposed were implemented for three different concentrations for the distinct samples, to evaluate the impact of concentration variations on the calculation of the mean nanoparticle diameter. The concentrations of 1:60, 1:80, and 1:100 mL, initially tested for samples S1, S2, and S3, respectively, were selected due to their favorable experimental conditions—exhibiting satisfactory scattering intensity levels and reduced noise in the data. Based on these preliminary results, concentrations near the optimal values were tested without modifying the previously established filtering parameters for each sample. The results of these additional tests are presented in Table 6, which displays the mean values calculated for each parameter before and after band-pass filter application. As in prior analyses, each of these values corresponds to the average of a set of ten consecutive measurements taken for each evaluated concentration.

**Table 6:** Results Obtained for Each of the Silica Samples Diluted at Different Ethanol Concentrations^a^

sample	concentration [mL]	⟨ρ_o_⟩ [s^–1^]	⟨ρ_f_⟩ [s^–1^]	⟨τ_c_(ρ_o_)⟩ [s]	⟨τ_c_(ρ_f_)⟩ [s]	⟨*d*_h_(ρ_o_)⟩ [nm]	⟨*d*_h_(ρ_f_)⟩ [nm]
S1	1:50	18.841	26.760	0.0207	0.0146	153.0	107.7
	1:60	34.891	23.360	0.0112	0.0167	82.9	123.3
	1:70	33.635	22.480	0.0116	0.0173	85.6	128.2
S2	1:60	35.641	14.329	0.0119	0.0272	87.9	201.1
	1:70	16.284	13.186	0.0247	0.0296	182.4	218.5
	1:80	26.561	12.176	0.0149	0.0320	109.7	236.6
S3	1:70	39.295	9.584	0.0100	0.0407	73.6	300.7
	1:80	28.920	8.592	0.0136	0.0454	100.5	335.3
	1:100	21.869	8.884	0.0179	0.0439	131.9	324.3

a The values corresponding to each of the listed parameters represent an average derived from a set of 10 consecutive measurements performed for each concentration.

For sample S1, in addition to the initial concentration of 1:60 mL, concentrations of 1:50 and 1:70 mL were also evaluated. The hydrodynamic diameters estimated after band-pass filter application showed relatively close values, suggesting that dilution variations fall within the optimal range for obtaining reliable and precise estimates. This behavior indicates good robustness of the MDSS method against small concentration variations, at least within the tested range.

A similar pattern was observed for the other samples: additional concentrations of 1:60 and 1:70 mL were tested for sample S2, and 1:70 and 1:80 mL were tested for sample S3. In all cases, the estimated mean diameters remained within a consistent range, demonstrating method stability for minor dilution changes.

Notably, however, the initially selected concentration for each sample systematically yielded the smallest estimated diameter among the tested concentrations. This behavior may be attributed to increased noise in less diluted samples, which promotes the appearance of significant artificial peaks in the scattering signal. As expected, these peaks reduce the estimated correlation time, consequently resulting in a smaller hydrodynamic diameter.

The plots in Figure 4, generated after applying the band-pass filter, illustrate the diameter variation as a function of time for each sample. Figure 4a–c displays the estimated diameters throughout the data set for the three tested concentrations, while Figure 4d shows the mean diameter value obtained across these concentrations. In each plot, the darker-toned curves represent the mean diameters calculated from the ten consecutive measurements performed for each concentration. The shaded regions around the curves indicate the associated standard deviation, reflecting the variation between the estimated results. The diameters highlighted in each figure, accompanied by their respective standard deviations, represent the estimated mean values for each concentration. These averages were calculated based on the final diameters obtained in the ten measurements performed per concentration, similar to what was presented in Tables 3–5. To ensure satisfactory and reliable estimates of the mean diameter of the samples, a minimum time of 30 s was adopted. This choice was based on the analysis of several measurements, which indicated stabilization of the results from this point on. This behavior can be clearly observed in the corresponding graphs.

**Figure 4:**
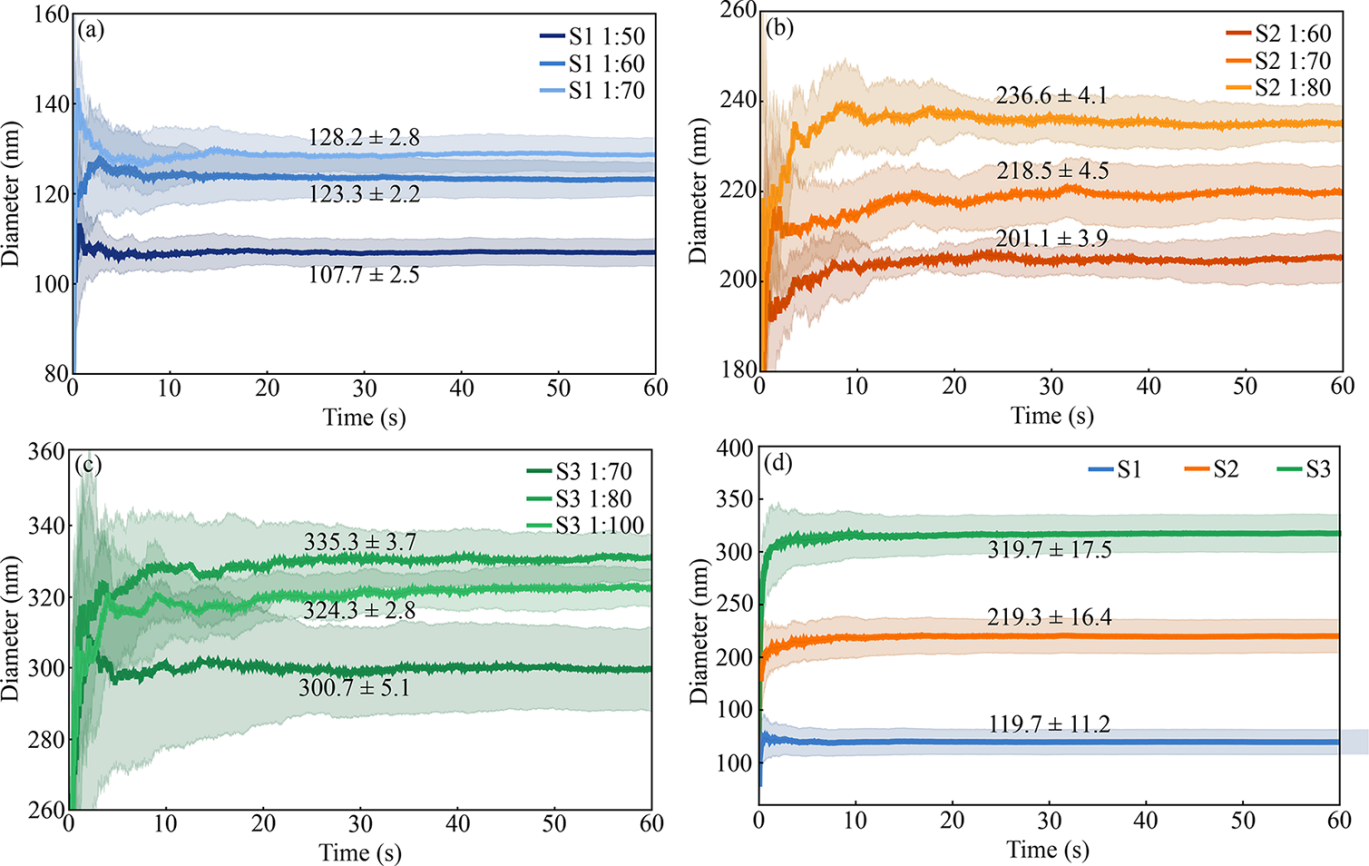
Diameter as a function of time for each silica sample. (a)–(c) Estimated diameters over time for the different tested concentrations. (d) Mean diameter values obtained across these concentrations for the three distinct samples. In each plot, the darker-toned curves represent the mean diameters calculated from the 10 consecutive measurements performed for each concentration. The shaded regions around the curves indicate the associated standard deviation, reflecting the variation between estimated results. The highlighted diameters, accompanied by their respective standard deviations, correspond to the average values obtained for each concentration, estimated based on the 10 measurements performed for each of them.

Analysis of the plots reveals that in all cases the curves initially exhibit greater variation in the estimated diameters, followed by stabilization after a certain time. This behavior is partially attributed to the methodology employed for sample diameter estimation, which was based on a progressive data analysis. During the initial stages of analysis, calculations are performed by using shorter time intervals that tend to contain fewer and more variable peaks in the scattering signal. This instability contributes to highly significant fluctuations in the initial estimates. As time progresses, the number of peaks per time interval stabilizes, yielding more consistent and closely aligned estimates.

Conversely, this initial behavior may also be associated with sample polydispersity. Figure 1, which displays an actual image of the silica nanoparticles, reveals some variation in particle diameters, supporting this hypothesis. Such heterogeneity can directly influence particle sedimentation dynamics and mobility over time. Larger particles tend to sediment more rapidly toward the lower regions of the cuvette, while smaller, more mobile particles contribute less significantly to dynamic light scattering. Consequently, particles with sizes closer to the mean eventually dominate the scattering signal as time progresses, resulting in a more substantial contribution to the scattering process.

Table 7 presents a direct comparison between the values previously estimated by conventional DLS and TEM techniques and those obtained via the MDSS method. The table includes the DLS estimates for samples S1 and S2, TEM estimates only for sample S1, and the values corresponding to sample S3, calculated exclusively based on the volume of NH_4_OH used in its synthesis. At the end, the silica and ethanol concentrations used in each sample are presented, along with the average diameters estimated for these concentrations.

**Table 7:** Direct Comparison between the Values Previously Estimated by Conventional Techniques and Those Obtained by the MDSS Method^a^

sample	diameter NH_4_OH [nm]	diameter DLS [nm] (conventional)	diameter TEM [nm]	concentrations used MDSS [mL]	diameter MDSS [nm] (concentrations average)
S1		146 ± 28	122 ± 50	1:50, 1:60, 1:70	119.7 ± 11.2
S2		226 ± 50		1:60, 1:70, 1:80	219.3 ± 16.4
S3	≈ 300			1:70, 1:80, 1:100	319.7 ± 11.2

a The second column presents the approximate diameter of sample S3, estimated exclusively based on the volume of NH_4_OH used in its synthesis. Columns three and four display the diameters previously determined by the conventional techniques of DLS and TEM, respectively. The fifth column indicates the concentrations of ethanol and silica used in the MDSS experiments. On the sixth column, the average diameters obtained considering the three different concentrations using the MDSS method. The results are in perfect agreement with the hydrodynamic diameters obtained from conventional DLS and TEM.

The analysis of the results reveals that the values obtained using the approach proposed in this study are quite close to those provided by traditional techniques. The consistency and precision achieved through the MDSS method were highly satisfactory, making this methodology an attractive alternative for particle characterization, especially when compared with methods that involve greater operational complexity and costs, as in the case of the TEM technique.

## Conclusions

The primary objective of this work was to investigate the feasibility of applying the density of maxima theory to dynamic light scattering experiments, specifically testing the maximum density speckle scattering technique. The density of maxima was originally developed for the statistical analysis of nuclear reactions and later extended to analyze conductance fluctuations in chaotic quantum dots. Its subsequent successful application in diverse contexts for determining correlation times in chaotic time series was a key factor in considering its applicability to DLS experiments, where the precise measurement of correlation time is of significant interest. The technique’s ability to derive distinct relationships for correlation times as a function of the density of maxima from the system’s characteristic correlation function enabled us to establish a relationship specifically tailored for DLS experiments, which constitutes one of the principal results presented in this study. Furthermore, the similarity between the scattering phenomena observed in DLS and the formation of dynamic speckles was essential to guide us in deducing a suitable relation for the correlation time, ensuring its direct applicability in DLS experiments.

Unlike conventional DLS, the MDSS technique does not require precise knowledge of optical parameters like refractive index or wavelength to calculate the scattering vector (*q*). This is a major limitation of DLS that MDSS overcomes. MDSS works by measuring the “clock speed”—the characteristic rate—of the scattered light’s intensity fluctuations caused by Brownian motion. This rate is directly related to the particle size. For a calibrated instrument, all the complex optical properties are folded into a single constant. Because MDSS uses this fluctuation speed instead of an absolute *q^2^* value, it should be robust for measuring particles in complex media where optical parameters are difficult to define.

The preliminary sample characterization, as outlined in the Materials and Methods section, was pivotal for generating comparative data that allowed us to assess the efficacy of MDSS. Concentration selection, along with the appropriate application of the noise filter on the Tektronix MSO2024B oscilloscope, was crucial for acquiring data with minimal noise under the given experimental conditions. However, despite these measures, a significant noise component remained. This residual noise led to considerable inaccuracies in the estimated mean particle diameters. To address this problem, we implemented a refined filtering process—using the autocorrelation function of the scattering signal—which proved effective in isolating noise and preserving the relevant signal information.

The MDSS method enabled estimates of the average diameter of silica nanoparticles with precision comparable to conventional techniques such as DLS and TEM. The measurements we performed at concentrations with lower noise levels—considered ideal for obtaining the initial results—exhibited remarkable consistency, yielding average diameter values that were extremely close to one another. Tests with concentrations close to ideal reinforced this pattern, evidencing the robustness and reliability of the method as a viable alternative for the characterization of suspended particles. As we have demonstrated, MDSS not only matches traditional approaches but also offers specific advantages—such as simplifying the characterization process by allowing the extraction of the correlation time directly from the chaotic time series of the scattering signal—proving to be a precise, efficient, and practical alternative for DLS experiments.

For future studies, several promising directions could be explored. First, it would be valuable to test the method with diverse sample types beyond those used in this study, employing varied materials and particle sizes to evaluate its performance across broader scenarios. Additionally, investigating new instrumentation capable of further enhancing scattering signal acquisition and ideally reducing residual noise in final data sets would be advantageous. The adoption of more compact equipment could enable the development of streamlined, potentially portable experimental setups suitable for field measurements.

This work makes significant contributions to both micro- and nanoparticle characterization and analysis of chaotic phenomena in dynamic systems. As we have hypothesized, the use of the maxima density to determine correlation times in a chaotic time series can be extended to various phenomena and applications. The present study has comprehensively demonstrated that the theory of maxima density, combined with the analysis of dynamic speckle formation, can be successfully applied in DLS experiments, enabling the precise determination of dimensions for particles under Brownian motion. This novel application not only expands data analysis possibilities in DLS but also provides a robust, effective solution to nanoparticle characterization challenges by proposing an alternative approach to conventional DLS methodologies.
